# Iterative Evolution of Sympatric Seacow (Dugongidae, Sirenia) Assemblages during the Past ∼26 Million Years

**DOI:** 10.1371/journal.pone.0031294

**Published:** 2012-02-03

**Authors:** Jorge Velez-Juarbe, Daryl P. Domning, Nicholas D. Pyenson

**Affiliations:** 1 Laboratory of Evolutionary Biology, Department of Anatomy, Howard University, Washington, D.C., United States of America; 2 Department of Paleobiology, National Museum of Natural History, Smithsonian Institution, Washington, D.C., United States of America; 3 Departments of Mammalogy and Paleontology, Burke Museum of Natural History and Culture, University of Washington, Seattle, Washington, United States of America; Raymond M. Alf Museum of Paleontology, United States of America

## Abstract

Extant sirenians show allopatric distributions throughout most of their range. However, their fossil record shows evidence of multispecies communities throughout most of the past ∼26 million years, in different oceanic basins. Morphological differences among co-occurring sirenian taxa suggest that resource partitioning played a role in structuring these communities. We examined body size and ecomorphological differences (e.g., rostral deflection and tusk morphology) among sirenian assemblages from the late Oligocene of Florida, early Miocene of India and early Pliocene of Mexico; each with three species of the family Dugongidae. Although overlapping in several ecomorphological traits, each assemblage showed at least one dominant trait in which coexisting species differed. Fossil sirenian occurrences occasionally are monotypic, but the assemblages analyzed herein show iterative evolution of multispecies communities, a phenomenon unparalleled in extant sirenian ecology. As primary consumers of seagrasses, these communities likely had a strong impact on past seagrass ecology and diversity, although the sparse fossil record of seagrasses limits direct comparisons. Nonetheless, our results provide robust support for previous suggestions that some sirenians in these extinct assemblages served as keystone species, controlling the dominance of climax seagrass species, permitting more taxonomically diverse seagrass beds (and sirenian communities) than many of those observed today.

## Introduction

The early evolution of sirenians parallels that of cetaceans, showing a transition from an amphibious to an obligate aquatic lifestyle [1]. Their herbivorous diet and low metabolic rate have mostly confined them over the last 50 million years to tropical and subtropical shallow-water habitats with abundant plant resources, and this together with high vagility likely restricted their evolutionary diversification [2]. Living representatives include two families, Trichechidae (manatees) with three species (*Trichechus manatus*, *T. senegalensis* and *T. inunguis*) and Dugongidae (dugongs) with two, *Dugong dugon* and *Hydrodamalis gigas* (the latter being the recently exterminated Steller's sea cow). Extant species are mostly allopatric, with dugongs occurring in the Indopacific region, whereas manatees are found along the Atlantic littoral zone and in freshwater systems that discharge into that ocean. The only exception is the parapatry or sympatry of *Trichechus manatus* and *T. inunguis*, which may overlap at the mouth of the Amazon River [3]. The fossil record of sirenians, in contrast, shows rampant sympatry [2]; coupled with their exclusively herbivorous diet, the occurrence of multispecies assemblages suggests that niche partitioning may have structured these ancient sirenian communities [4].

Following Domning [2] and Velez-Juarbe and Domning [5], we considered a suite of morphological features as ecomorphological proxies, including: (1) tusk (upper first incisor) morphology (size and cross-sectional shape); (2) rostral deflection; and (3) body size [see Materials and Methods for further explanations of each parameter]. We then applied these proxies in three different sympatric dugongid assemblages from the late Oligocene (Florida), early Miocene (India), and early Pliocene (Mexico). Because these proxies are directly tied to sirenian dietary and foraging preferences, this comparative study elucidated major features of sirenian ecology that have repeatedly evolved in the geologic past, and in separate ocean basins. Moreover, the presence of multispecies sirenian communities until the Pliocene suggests that ancient seagrass community ecology differed from today's in both diversity and energy flow patterns, with extinct dugongids possibly serving as keystone species.

## Materials and Methods

### Institutional abbreviations

**ECOCHM**, Museo de Zoología, El Colegio de la Frontera Sur (ECOSUR), Chetumal, Quintana Roo, Mexico; **SC**, South Carolina State Museum, Columbia, South Carolina, U.S.A.; **UF**, Florida Museum of Natural History, University of Florida, Gainesville, Florida, U.S.A.; **UF/FGS**, former Florida Geological Survey collection, now housed at the Florida Museum of Natural History.

### Localities & geological ages

The three assemblages examined here each included at least three dugongid species, belonging to the subfamilies Halitheriinae or Dugonginae. Information about fossil taxa in each assemblage, outlined below, was obtained from the literature and direct observations of each specimen. Information about UF 49051, ECOCHM 2488 and ECOCHM 2491 represents previously unpublished data.

#### Florida

Beds of the Parachucla Formation of the Hawthorn Group are exposed along the Suwannee River, near White Springs, Hamilton County, Florida [6], [7]. One locality, about 300 meters north of the U.S. Highway 41 bridge, yielded two sirenian skulls, one belonging to *Dioplotherium manigaulti* (UF 95615), the other (UF 49051) to a yet-undescribed species of *Metaxytherium* [6], [8]. A second locality, about 2.6 km west of White Springs, yielded the holotype skull of *Crenatosiren olseni* [7], [9]. Both deposits seem to represent the same bed [7]. The age of the locality where UF 49051 and UF 95615 were collected was originally regarded as early Miocene, but then later considered as late Oligocene [6], [7]. *Crenatosiren olseni* is also known from the late Oligocene (25.7–23.6 Ma [10]) Chandler Bridge Formation of South Carolina [7]. This formation has also yielded remains of *Metaxytherium* very similar to UF 49051, and likely of the same undescribed species (JVJ & DPD, personal observations of SC 89.115). The similarity in the sirenian taxa between these two formations is consistent with a late Oligocene age for the Florida localities.

#### India

The localities in India are all in the district of Kutch, Gujarat State, western India, where the Aquitanian or Burdigalian (early Miocene) Khari Nadi Formation is exposed [11]–[13]. Three dugongine dugongids from three localities, about 12–15 km apart, are known from this formation: *Bharatisiren kachchhensis*, *Domningia sodhae* and *Kutchisiren cylindrica* [11]–[13]. Although these were collected from different beds within the same formation, we consider it likely that these species represented sympatric lineages and treat them as such.

#### Mexico

The Mexican locality is about 1 km SSW of Km 40.5 on the road from Tizimin east to Colonia Yucatan, in the State of Yucatán, Mexico [14]. Here, the early Pliocene (5.3–3.6 Ma) Carrillo Puerto Formation is exposed on the ground surface; two sirenian skulls and a mandible were collected at this locality, within about 7 m of each other. Only one of the skulls (IGM 4569) has been described so far, as *Corystosiren varguezi* [14], whereas the other skull (ECOCHM 2491) and mandible (ECOCHM 2488) remain the focus of future descriptive work. Preliminarily, these specimens represent *Nanosiren* sp. cf. *N. garciae* (ECOCHM 2488) and an undescribed species of *Dioplotherium* (ECOCHM 2491). Another possibly co-occurring sirenian in this formation and age was *Xenosiren yucateca* [15]. Fragmentary remains of this taxon indicate that it possessed specializations to uproot very large seagrass rhizomes, despite being less adept at processing fibrous material [2], [15]. Its presence would imply at least four sympatric sirenians in the early Pliocene of Mexico, but the incompleteness of this single specimen, coupled with doubts about the age of its horizon, preclude us from making definitive inferences about its paleoecology [14], [15].

### Phylogenetic analysis

We conducted a phylogenetic analysis of the relationships among the dugongids studied herein using a new character-taxon matrix based primarily on Domning and Aguilera [16], along with some modifications (see Text S1). The resulting matrix included 43 characters for 34 terminal taxa, including *Moeritherium* (Proboscidea) and *Paleoparadoxia* (Desmostylia) as basal outgroups (following Domning [17]). We analyzed our matrix using TNT (Tree Analysis Using New Technology [18]). Characters were set as unordered. We performed a traditional search using the tree bisection-reconnection (TBR) swapping algorithm, with the following settings: 1000 replicates, keeping 10 trees per replicate.

### Ecomorphological categorization and body size estimation

#### Tusk Morphology

**Figure 1:**
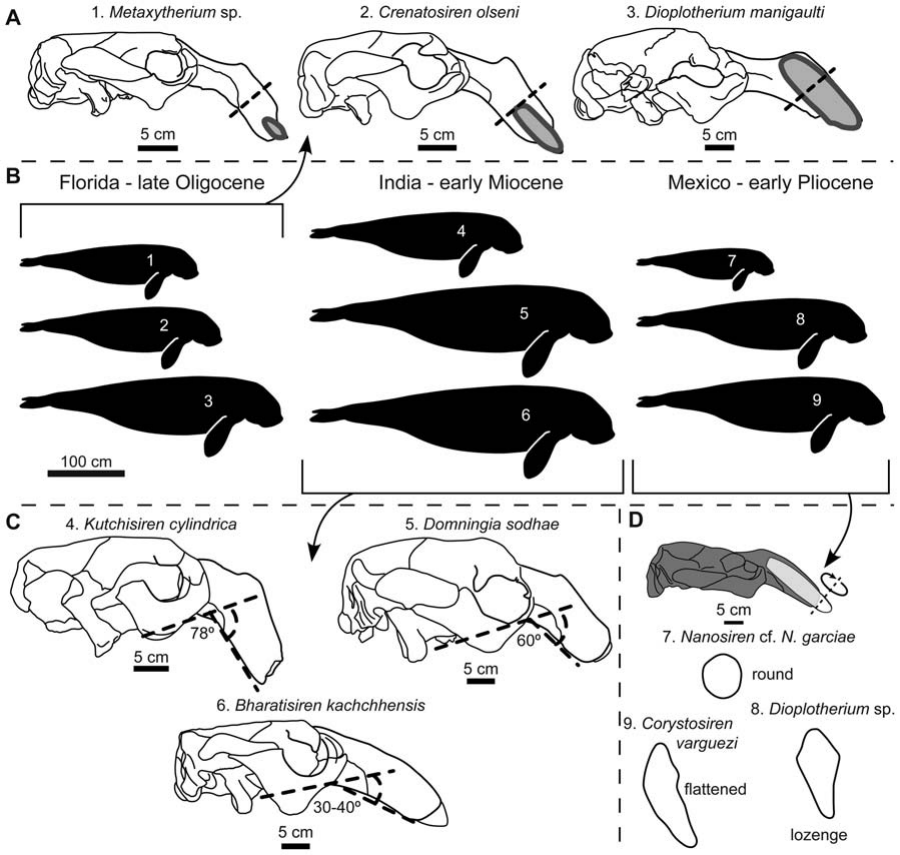
Sirenian taxa and ecomorphological features used in this study. A) Fossil dugongids from the late Oligocene Parachucla Formation of Florida showing categories of upper incisor 1 alveolar size and depth (outlined in gray), compared to premaxillary symphysis length (dashed line denotes mid-length). *Metaxytherium* sp. based on UF 49051, *Crenatosiren olseni* and *Dioplotherium manigaulti* modified from Domning [9] and [8], respectively. B) Comparison of body sizes among dugongids used in this study. C) Fossil dugongids from the early Miocene Khari Nadi Formation of India showing differences in rostral deflection. Illustrations modified from [10]–[12]. D) Cross-sectional outline of incisors of fossil dugongids from the early Pliocene Carrillo Puerto Formation of Mexico. Skull of *Corystosiren varguezi* (modified from [11]) shown to demonstrate tusk cross-section.

It has been hypothesized that the tusks of extinct sirenians were used as tools for uprooting seagrass rhizomes, with tusk size and shape positively correlated with the maximum size of the rhizomes that each species could have uprooted [2], [4]. Isotopic analyses have also supported this correlation [19]. In this study, we divided tusk and alveolus size into three categories, based on the depth of the alveolus relative to the premaxillary symphysis (Figure 1A) [17]. Tusk shape referred to its outline in cross-section, which we divided into: round or suboval; lozenge-shaped; and extremely flattened mediolaterally (Figure 1D) [2].

#### Rostral Deflection

All sirenians have rostra that are, to varying degrees, ventrally deflected. Although the role of rostral deflection is incompletely studied, as Domning [2] indicated, it plays a role in bottom-feeding. A greater degree of rostral deflection positions the mouth closer to the seafloor while keeping the rest of the body nearly horizontal, thus facilitating horizontal swimming while feeding. Horizontal body position would also have facilitated feeding at depths of <1 m, by allowing bottom-feeding without having to lift the tail out of the water. We measured rostral deflection as the angle formed between the rostral palatal plane and the maxillary occlusal plane (Figure 1C). For specimens lacking crania, we took this measurement from the mandible in a similar fashion, as these angles are nearly the same (except for *Kutchisiren cylindrica* and *Dioplotherium* cf. *Di. allisoni* [13], [20]).

#### Body size

Body size in marine mammals, like other organisms, strongly bears on their ecological role (see discussion in [21]). Large body size in sirenians, among other advantages (e.g., predation deterrence), would permit more efficient processing of larger rhizomes (especially because sirenians are hindgut fermenters [22]), but it would also require larger foraging areas and restrict foraging to deeper water. Smaller sirenians would be able to reach and exploit resources in shallower areas where larger ones would run the risk of stranding [16]. Body size estimates were calculated using Sarko et al. [23]'s equation 1, which uses skull condylobasal length as a proxy. This equation is based on measurements of *Du. dugon*, which is the extant relative phylogenetically closest to the fossil taxa in this study.

## Results

**Figure 2:**
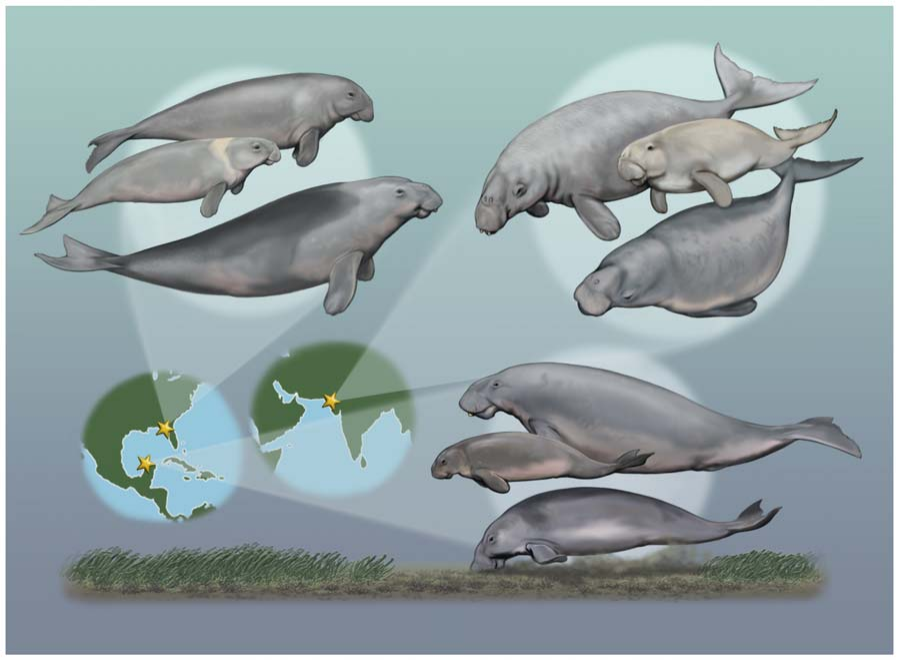
Illustration showing reconstructions of fossil dugongid assemblages from Florida, India and Mexico during the past ∼26 million years. Clockwise from the top left, the geographic location of each assemblage is denoted by a gold star, and includes the following taxa, arranged from top to bottom in each spotlight: Florida: *Crenatosiren olseni*, *Metaxytherium* sp., and *Dioplotherium manigaulti*; India: *Bharatisiren kachchhensis*, *Kutchisiren cylindrica*, and *Domningia sodhae*; Mexico: *Corystosiren varguezi*, *Nanosiren* cf. *N. garciae*, and *Dioplotherium* sp., which is depicted feeding on seagrasses. Art by Carl Buell.

Unlike modern sirenians, some fossil sirenian species were sympatric. Overall, our study demonstrated how discrete ecomorphological proxies available from the fossil record can differentiate among co-occurring, yet phylogenetically independent sirenian taxa in fossil assemblages that have existed at different times and in different depositional basins over the past ∼26 million years (Figure 2).

**Figure 3:**
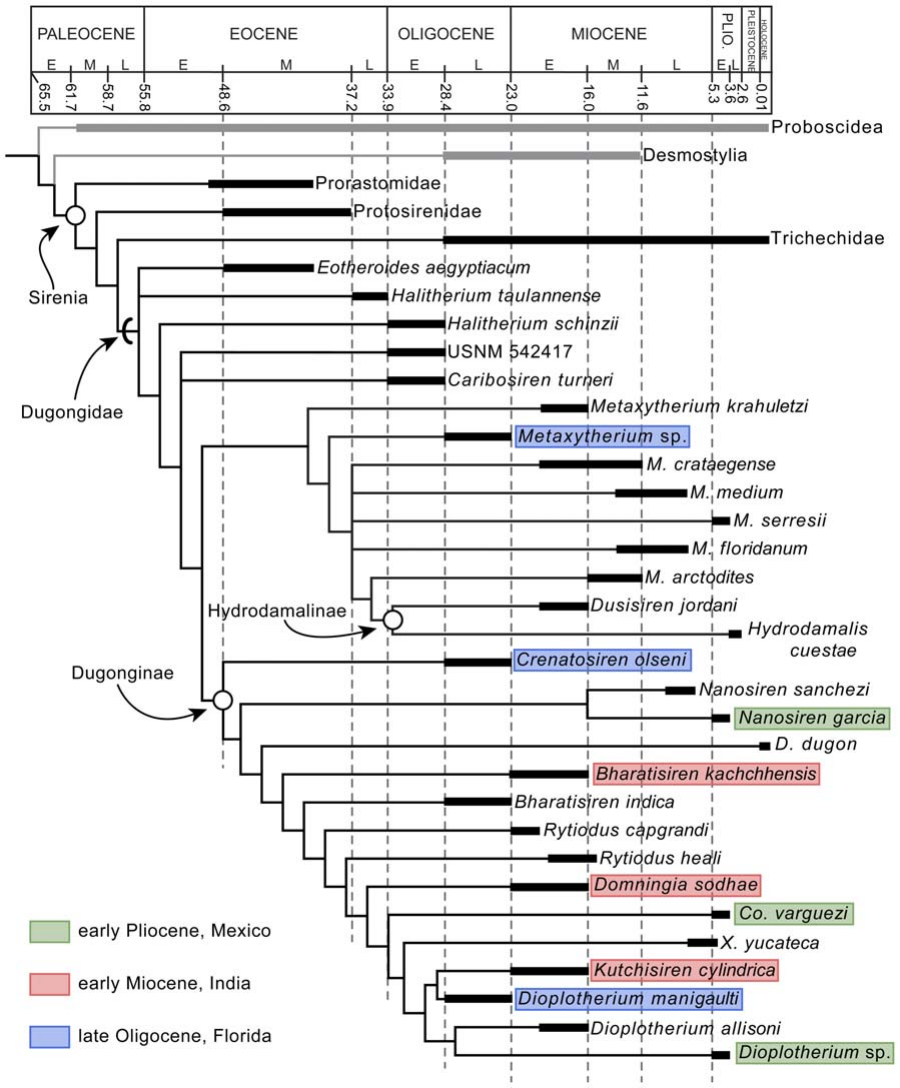
Time-calibrated phylogeny of Sirenia. Consensus tree (length = 127; ri = 0.758; ci = 0.527). Taxa included in this study are highlighted in color, as follows: green, early Pliocene of Mexico; red, early Miocene of India; blue, late Oligocene of Florida. Open circles identify node-based clades; arcs indicate stem-based taxa. The poorly integrated group “Halitheriinae” is paraphyletic in this study.

Our phylogenetic analysis resulted in two most parsimonious trees, which were each 126 steps long. The strict consensus tree (Figure 3) is 127 steps long and has a consistency index (CI) = 0.53 and retention index (RI) = 0.76. Our results are largely consistent with previous phylogenetic analyses of fossil sirenians [16], [17], although it differs in recovering a monophyletic Dugongidae, in contrast to Domning [17], which posited a paraphyletic Dugongidae, with Trichechidae nested within it. Within Dugongidae, we recovered a paraphyletic “Halitheriinae,” and monophyletic Hydrodamalinae and Dugonginae. This analysis revealed that the dugongid taxa belonging to the assemblages targeted in this study were largely unrelated to one another; none of the taxa in any of the assemblages (even congeneric ones) were sister taxa. For example, in the oldest assemblage, from the late Oligocene of Florida, *Metaxytherium* sp. was included within a clade with other species of that genus and with Hydrodamalinae, whereas the other two taxa, *Crenatosiren and Dioplotherium manigaulti*, were the basalmost and one of the more derived members, respectively, of the Dugonginae (Figure 3). The Indian and Mexican dugongids were all recovered within Dugonginae. Among the Indian dugongines, *Bharatisiren kachchhensis* is one of the most basal members of the subfamily, whereas *Domningia* and *Kutchisiren* are more derived and more closely related to taxa from the Western Atlantic and Caribbean region. Like the Indian assemblage, the Mexican assemblage also had a basal-branching species (i.e., *Nanosiren*), whereas the other two were more derived.

Each fossil assemblage demonstrated clear partitioning among co-occurring dugongids by at least one dominant trait. First, the Oligocene dugongids from Florida differed in body size and tusk morphology (Figure 1A, 1B, Table 1). The two taxa with the most similar body sizes (*Crenatosiren olseni* and *Metaxytherium* sp.) differed in having medium and small tusks, respectively, whereas the larger (by nearly 1 meter) *Di. manigaulti* had large tusks (Figure 1A). The relatively small body sizes of *Metaxytherium* sp. and *C. olseni* likely conferred an ability to forage in shallower waters than *Di. manigaulti*. Furthermore, different tusk sizes likely represented differences in ability to uproot seagrass rhizomes of larger sizes, thus minimizing competition for food amongst each other even when foraging in the same area [4]. In this assemblage, *Metaxytherium* and *Crenatosiren* likely preferred small and medium-size rhizomes, whereas *Di. manigaulti*, with its large tusks, was better suited to uproot much larger rhizomes. Congruent specializations can be observed in their cranial architecture, with the premaxilla/frontal contact in *Di. manigaulti* forming a butt joint, in contrast to the more overlapping contact seen in *Crenatosiren* and *Metaxytherium* (Figure S1). A butt joint contact between premaxilla and frontal has been proposed as an osteological adaptation against stresses resulting from uprooting very large, deeply-buried seagrass rhizomes [2], [4].

**Table 1:** List of sirenian taxa and categorization of corresponding ecomorphological features used in this study.

Taxa	Rostral deflection (degrees)	Estimated body size (cm)	Depth and size of tusk (I1)^a^	Tusk (I1) shape (cross section)
**Late Oligocene – Florida**				
1. *Crenatosiren olseni*	55	236	Medium	Suboval
2. *Metaxytherium* sp.	60	267	Small	Round
3. *Dioplotherium manigaulti*	50–55	350	Large	Lozenge
**Early Miocene – India**				
4. *Kutchisiren cylindrica*	78	282	Large	Lozenge
5. *Domningia sodhae*	60	402	Large	Lozenge
6. *Bharatisiren kachchhensis*	30–40	404	Large	Suboval
**Early Pliocene – Mexico**				
7. *Nanosiren* cf. *N. garciae*	68	183	Small	Round
8. *Dioplotherium* sp.	30–40	306^b^	Large	Lozenge
9. *Corystosiren varguezi*	30–40	331^c^	Large	Flattened

a **Small**, length less than half the length of the premaxillary symphysis; **medium**, length about half the length of the premaxillary symphysis; **large**, length greater than half the length of the premaxillary symphysis.

b Estimate based on condylobasal length of skull of 41.5 cm.

c Based on *Nanosiren garciae* with which it shares similar dimensions of the mandible.

All of the Indian species had large tusks, but they differed in body size and rostral deflection (Figure 1B, 1C, Table 1). *Domningia sodhae* and *Bharatisiren kachchhensis* had similar body sizes, both being larger, by at least 1 meter, than *Kutchisiren cylindrica* (Figure 1B, Table 1). The overlap in both body and tusk size between *Do. sodhae* and *B. kachchhensis* might reflect similarities in feeding preferences as well as foraging areas; however, their cranial morphology suggests otherwise. Like *Dioplotherium*, the premaxilla-frontal contact in *Domningia* forms a butt joint (DPD, pers. obs.), whereas *Bharatisiren* shows a long tapering overlap [11]. Thus, *Domningia* likely uprooted the largest rhizomes, whereas *Bharatisiren* might have done this to a lesser extent, even with its large tusks. *Kutchisiren cylindrica*, the smallest of the three Indian species, showed the greatest rostral deflection (Figure 1A). Its shallower draft would have allowed it to forage in shallows inaccessible to the other species. In addition, its greater rostral deflection permitted bottom-feeding in very shallow water while keeping its body in a nearly horizontal position, without having to lift the tail out of the water.

The Mexican sirenians showed more similarities with the Indian assemblage than with the one from Florida, despite large temporal and geographic separation. In the Mexican assemblage, there were also two dugongid species with large body size, and one (*Nanosiren*) at least 1 meter smaller than the others (Figure 1B, 2, Table 1). Known species of *Nanosiren* have small, round tusks [16], although this particular element was missing from the Mexican specimen of *Nanosiren*. In contrast, *Dioplotherium* sp. and *Corystosiren varguezi* had large tusks with differing cross-sectional outlines (Figure 1D). *Corystosiren*'s more blade-like tusks were probably better at harvesting larger seagrass rhizomes than the lozenge-shaped tusks of *Dioplotherium* [4]. Therefore, based on tusk and cranial morphology, *Nanosiren* likely fed on small seagrasses, whereas *Dioplotherium* sp. and *Corystosiren* fed on large and very large seagrasses, respectively. In terms of foraging areas, *Nanosiren* (like *Kutchisiren*) was very likely able to forage in shallower water than its contemporaries, with its greater rostral deflection allowing horizontal trim while feeding and moving forward.

## Discussion

### Resource partitioning in extinct dugongid communities

Our results show that multispecies sirenian communities evolved repeatedly since the Oligocene in separate tropical and subtropical shallow seas, with each iteration involving phylogenetically unrelated members of the family Dugongidae (Figures 1, 2, and 3). Among the assemblages studied here we observed two overall patterns. First, in the assemblage from Florida, tusk morphology was the dominant trait for separating feeding preferences, with body size classes then providing an additional level of separation. In contrast, for both the Indian and Mexican assemblages, no single trait clearly differentiated all the taxa; rather, multiple ecomorphological traits separated co-ocurring dugongids. Based on our results (Table 1), we hypothesize that small-bodied taxa in these assemblages were generally associated with greater rostral deflection, which would have been advantageous for foraging in shallow waters (∼1 meter depth). This combination of small body size and strong rostral deflection evolved independently in the Indian and Mexican assemblages (which represent two different time periods and separate geographical regions). It remains to be tested whether this independent evolution reflects differences in seagrass community structure after the Oligocene, or, more plausibly, the result of selective pressures from competition with other sympatric sirenians as yet undiscovered. For larger-bodied taxa from these two assemblages, tusk morphology was more important than rostral deflection, which we propose played a minor role, when overshadowed by the dominance of the other traits in structuring these communities. Although rostral deflection is correlated with bottom-feeding to a first approximation, Domning [2] and Domning and Beatty [4] suggest that this ecomorphological trait is more complex: deflection may be reduced in a species that typically feed by staying in one place and vigorously digging a pit, rather than making a feeding trail while swimming continuously forward.

Generally, the structure of sympatric communities has been explained with interspecific competition as a fundamental evolutionary driver [24], [25], and the ecomorphologic traits used here, along with body size estimates, are consistent with such competition as a means of resource partitioning among sympatric sirenians. The iterative evolution of such resource partitioning has been inferred on an ecomorphological basis for fossil terrestrial mammal communities (e.g. [26], [27]), and recently for fossil sea turtles as well [28]. These results document this pattern for the first time in fossil marine mammal assemblages. Because our results showed that the taxonomic components of each assemblage involved taxa that were largely unrelated to one another (i.e., none were sister taxa), we argue that the iterative evolution of multispecies dugongid communities demonstrates the merit of extending questions about generality of community assembly [29], [30] into the fossil record of marine mammals, paralleling ongoing efforts with the comparatively denser and better-studied fossil record of terrestrial mammals [31]–[38]. Although we relied on ecomorphologic and body size proxies to detect the resource partitioning described for fossil dugongid communities, the application of other methods could possibly yield additional information. For example, stable isotope and tooth wear methods have added further evidence on the structure of extinct terrestrial mammalian communities [39]–[41], and we foresee promise in similar applications for studying fossil marine mammal communities as well (e.g. [19]).

Multispecies sirenian assemblages likely existed prior to the late Oligocene, for example in the middle to late Eocene of Egypt [42]. However, we excluded such data from our analyses because at least one of the parameters used in this study (i.e., body size estimates generated from *Dugong* data [23]) might be inapplicable to Eocene sirenians, some of whose bauplans still included hind limbs and possibly even amphibious lifestyles (e.g. *Protosiren smithae* [43]). At the coarsest level, our study assessed sirenian occurrence data at the formational level (and at the finest, by locality), a bracketing that we view as providing the most compelling basis for validating the association of these fossil dugongids with their source communities. In some cases, we expect that additional field collections from fossil marine mammal-bearing rock units will increase the known richness of multispecies sirenian assemblages (see Text S1).

In contrast to the results presented in this study, monotypic sirenian occurrences have been reported from well-sampled sequences in the Mio-Pliocene strata of the western margin of North America [44] and Tethys [19]. In both cases, these occurrences represent single (and arguably anagenetic) lineages that shifted dietary specializations in tandem with geological and environmental events that altered the marine flora in these two regions [16], [44]. In light of our results, we view such monotypic sirenian sequences as the exception, in contrast to multispecies assemblages, which we argue are more typical across the globe from the late Paleogene and Neogene. The youngest assemblage considered here, from Mexico, indicates that multispecies sirenian communities persisted until relatively recently (4–5 Ma), whereas a single sirenian species (*Trichechus manatus*) lives in the same area today. Thus, from a deep historical perspective, we argue that Holocene sirenian community diversity is aberrantly depauperate and allopatric, compared with the typical condition since the late Oligocene.

### Implications for the evolution and extinction of modern seagrass communities

As strict herbivores, sirenians are strongly tied to their resources, and, in all likelihood, seagrass diversity and ecology were both directly influenced by multispecies consumer communities in the geologic past. The ecological association between sirenians and seagrasses has at least a middle Eocene antiquity [45]–[47], although direct associations of their fossils are rare. The oldest known seagrasses are mid-Cretaceous [48], [49], but their fossil record is otherwise sparse. In the absence of a better fossil record of this plant resource, the fossil record of sirenians can serve as a proxy chronicle to constrain hypotheses about the evolution of seagrass communities [50]. Although there are late Neogene exceptions to this relationship – i.e., certain sirenian lineages have broadened (e.g., *Trichechus* spp. [51]) or changed (*Hydrodamalis* spp. [44]) their feeding preferences – we see several predictions that follow from this line of argument. For example, in terms of energy flow, we would predict that a larger portion of seagrass community primary productivity would have been processed by the expanded number and size range of consumers, rather than directly becoming detritus, as mostly happens today [52], [53]. Also, because the dugongids in each assemblage were each morphologically suited to consume differently sized seagrasses [2], [19], we hypothesize that the more ecologically important taxa in these extinct communities were large-tusked dugongids (e.g., *Dioplotherium*), which would have acted as keystone species [54]. We propose that large-tusked dugongids kept diverse seagrass communities from progressing to a climax state dominated by large species (e.g. *Thalassia*), and made room for smaller, pioneer species (e.g. *Halodule* and *Syringodium*) [2]. Lastly, because our phylogenetic analysis revealed that the lineages of each dugongid community member were unrelated to one another, we argue that the assembly of large marine herbivore consumer communities in the geologic past might have arisen from interspecific competition for the diverse seagrass resources, rather than shared genealogical history. In other words, our results suggest that competition for a unique resource has been the primary driver for the iterative evolution of specific morphotypes and body size classes in dugongids.

During the Cenozoic, large-scale changes in ocean circulation and global temperature would have most directly affected the evolution of large marine consumers [55]–[57], although the specific evolutionary signals for such changes, read from the records of changes in taxonomic diversity, are difficult to interpret [58], specially given the possible biases in the record of some groups [59]. Following a preliminary comparison by Uhen and Pyenson [59], Marx [60] found that sirenian richness through time did not parallel European richness of cetaceans or pinnipedimorphs (while accounting for possible biases, such as rock outcrop area), which suggested that the peaks and drops in richness of the sirenian fossil record likely reflect secular biological signals. Because sirenian are intimately tied with nearshore habitats (specially epicontinental surfaces), it is possible that their richness reflects the effects of eustatic sea-level change. For the peak in sirenian richness, we hypothesize that the flooding of continental platforms in the early to middle Miocene [61] increased available seagrass habitat, opening new opportunities for ecological diversification (see Hamilton et al. [62] for similar drivers in a scenario with Amazonian cetacean evolution). Our study was limited to three snapshots through time, which makes it difficult to test if multispecies communities of dugongids were related to specific climatic and oceanographic conditions. Further sampling, through additional field and museum work, should better resolve the relationship between changing Cenozoic environments and sirenian diversity.

Causes for the demise of multispecies sirenian communities remain unclear, but in the Western Atlantic and Caribbean (WAC), regional changes in the seagrass communities may have played a role. The sparse fossil record of seagrasses minimally sets a middle Eocene antiquity to their presence in the WAC [45], [49], [63]. Notably, the diversity of this middle Eocene assemblage is more similar, in richness, to extant Indo-West Pacific communities than to those currently found in the WAC [63]. Though no other fossil seagrasses are known from the WAC, the persistence of multispecies sirenian communities until at least 4–5 Ma suggests that Neogene seagrass communities were still richer than they are today. A major extinction event ∼2 Ma, after the closure of the Central American Seaway [64], might have eliminated some of the seagrasses, with a timing that is consistent with the last occurrence of dugongids in the region [2].

## Supporting Information

Figure S1 **Dorsal views of skulls of fossil dugongids from the Late Oligocene of Florida, showing the configuration of the premaxilla-frontal suture.** Anterior to the right.
(TIF)

Text S1 **Information on other potential sirenian multispecies assemblages; characters and matrix used in the phylogenetic analysis.**
(DOC)
