# Supplementary figures and images for: Iterative Evolution of Sympatric Seacow (Dugongidae, Sirenia) Assemblages during the Past ∼26 Million Years
Source: PLoS One. 2012 Feb 3;7(2):e31294. doi: 10.1371/journal.pone.0031294 (PMC3272043; doi:10.1371/journal.pone.0031294)

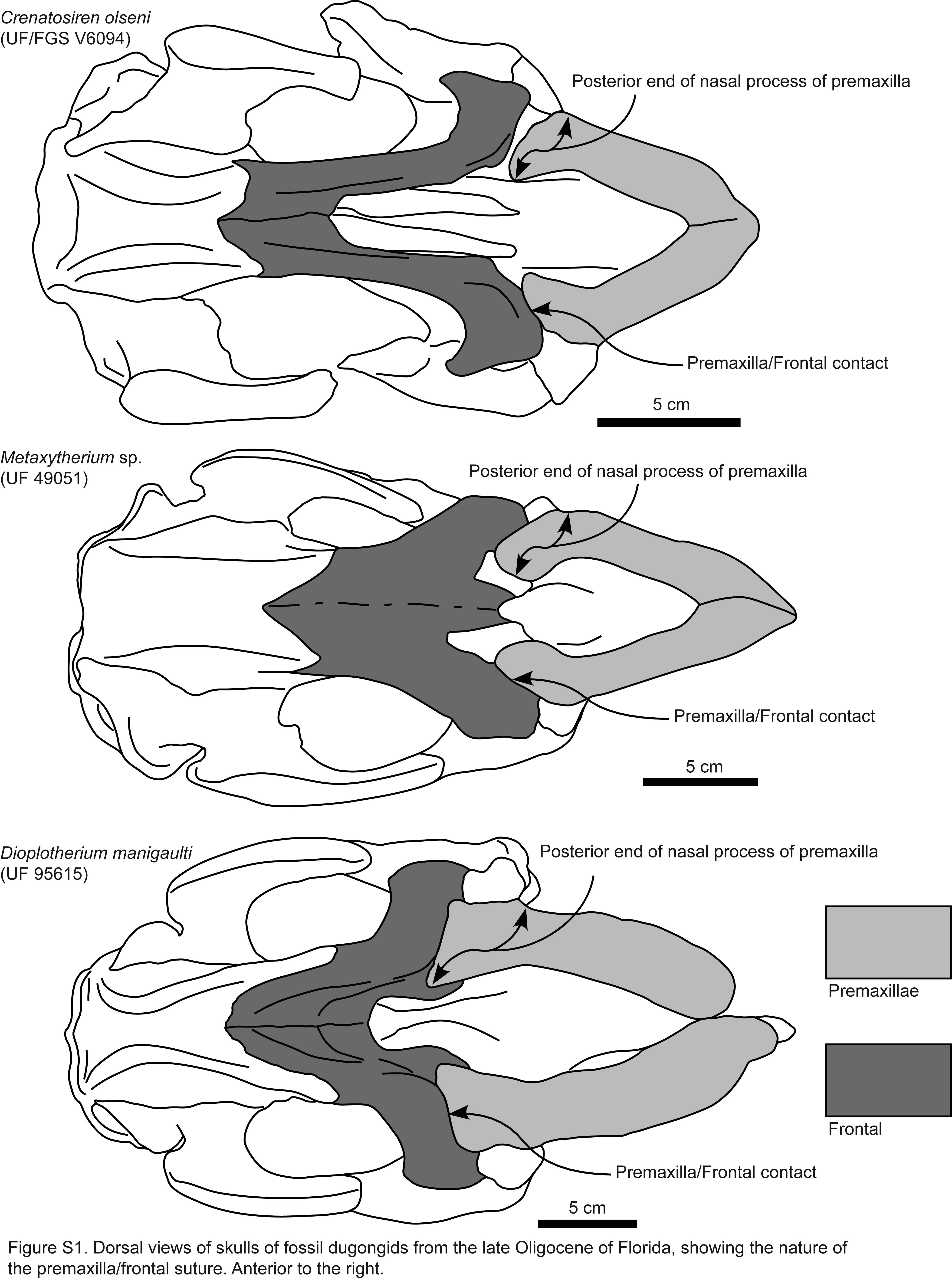

Supplement: Figure S1 — Dorsal views of skulls of fossil dugongids from the Late Oligocene of Florida, showing the configuration of the premaxilla-frontal suture. Anterior to the right. (TIF) [file pone.0031294.s001.tif]
