# Supplementary material for: Iterative Evolution of Sympatric Seacow (Dugongidae, Sirenia) Assemblages during the Past ∼26 Million Years
Source: PLoS One. 2012 Feb 3;7(2):e31294. doi: 10.1371/journal.pone.0031294 (PMC3272043; doi:10.1371/journal.pone.0031294)
Supplement: Text S1 — Information on other potential sirenian multispecies assemblages; characters and matrix used in the phylogenetic analysis. (DOC) [file pone.0031294.s002.doc]

Electronic Supplementary Material (ESM) for:

**Iterative evolution of sympatric seacow (Dugongidae, Sirenia) assemblages during the past ~26 million years**

**Jorge Velez-Juarbe^1,2^*, Daryl P. Domning^1,2^ and Nicholas D. Pyenson^2,3^**

^1^*Laboratory of Evolutionary Biology, Department of Anatomy, Howard University, Washington, DC 20059, USA*

^2^*Department of Paleobiology, National Museum of Natural History, Smithsonian Institution, Washington, DC 20560, USA*

^3^*Departments of Paleontology and Mammalogy, Burke Museum of Natural History and Culture, University of Washington, Seattle, Washington 98195, USA*

**Author for correspondence (velezjuarbe@gmail.com).*

**Text S1:** Information on other potential sirenian multispecies assemblages; characters and matrix used in the phylogenetic analysis.

Table of Contents:

1. Other Assemblages p. S2
2. Phylogenetic Analysis p. S2
3. Matrix p. S7
4. References for Supplementary Material p. S10

**1. Other potential multispecies assemblages**

Other possible Neogene instances of multispecies dugongid assemblages include the early Miocene of Europe, Libya, Panama and Brazil [1-4]; the middle Miocene of California and Baja California and the Chesapeake region of the eastern USA [5-7]; and the early Pliocene of Florida [8]. However, in these deposits, some of the taxa and/or stratigraphy are still very poorly known. We decided not to evaluate pre-Oligocene assemblages, even though they do exist (e.g. the Eocene of Egypt), because at least one of the parameters used in this study (body size estimates generated from *Dugong* data [9]) might be inapplicable to primitive sirenians whose bauplan still included hind limbs, and possibly even amphibious lifestyles (e.g. *Protosiren smithae* [10]).

All of the specimens used in this study are adult individuals, and with the exception of *Crenatosiren olseni* and *Domningia sodhae*, all the species are known only from single specimens. Furthermore, we are certain that, during life, sympatric taxa were occupying the same general areas, as their distribution would be constrained by that of the seagrasses (their principal food source): mainly shallow, protected marine environments [11].

**2. Phylogenetic Analysis**

The phylogenetic analysis follows that published in Domning and Aguilera [8] with the addition of 11 characters. Of these, characters 3, 8, 9, 11, 101, 103, and 139 were added from Domning [12]; characters 64 and 147 were added from Bajpai et al. [13] and Domning et al. [14], respectively; character 43 was combined with character 36, following Bajpai et al. [13]. Characters 22 and 105 are newly introduced.

3. *Rostrum*: (0) Premaxillae do not form an elongated rostrum; (1) rostrum elongated but small relative to cranium (length of premaxillary symphysis smaller or equal to 0.27 x condylobasal skull length); (2) rostrum enlarged (length of premaxillary symphysis larger than 0.27 x condylobasal skull length).

6. *Nasal process of Premaxilla*: (0) thin and tapering at posterior end, overlapping frontal and/or nasal; (1) tapering but broadened at posterior end, with long overlap of frontal and/or nasal; (2) tapering but thickened at posterior end, with long overlap of frontal and/or nasal; (3) Broadened and bulbous at posterior end, having more or less vertical, transverse butt joint surface in contact with frontal and/or nasal

7. *Nasal process of Premaxilla*: (0) Long (distance from rear of symphysis to posterior end of premaxilla >1/2 length of symphysis); (1) Very short (distance from rear of symphysis to posterior end of premaxilla <1/2 length of symphysis).

8. *External nares*: (0) not retracted; (1) retracted and enlarged, reaching to or beyond the level of the anterior margin of orbit.

9. *Premaxilla*: (0) short, does not contact frontal; (1) long, contacts frontal; (2) long, contact with frontal nearly lost, premaxilla abuts mainly against nasal instead.

10. *Premaxillary symphysis*: (0) not laterally compressed to form a mid-dorsal ridge; (1) laterally compressed, bearing a mid-dorsal ridge anteriorly with its posterior end upraised to form a boss in lateral view; (2) laterally compressed but without a boss, posterior end being dorsally flattened instead.

11. *Zygomatic-orbital bridge of maxilla*: (0) nearly level with palate; (1) elevated above palate, with its ventral surface lying >1 cm above the alveolar margin.

14. *Zygomatic-orbital bridge of maxilla*: (0) long anteroposteriorly (vertical thickness < 0.40 x minimum length); (1) shortened (thickness greater than or equal to 0.40 x length); (2) shortened and transformed into a transverse vertical wall.

16. *Palate*: (0) thin or incomplete at level of penultimate cheek tooth; (1) > 1 cm thick at level of penultimate tooth.

20. *Infraorbital canal*: (0) not obstructed; (1) partly obstructed by a transverse bony ridge.

22. *Zygomatic-orbital bridge of maxilla*: (0) anterior edge thin and sharp, posterior edge thick and more or less rounded; (1) both edges thin and sharp; (2) both edges thick & rounded.

36. *Supraorbital process of frontal*: (0) well developed, dorsoventrally thin overall (< 2 cm thick) with prominent, dorsoventrally flattened posterolateral corner; (1) dorsoventrally thickened (more than 2 cm thick), with posterolateral corner only moderately or weakly developed; (2) reduced, rounded, lacking posterolateral corner; (3) dorsoventrally thickened (more than 2 cm thick), with dorsal surface inclined strongly ventrolaterad and with posterolateral corner strongly developed and more or less recurved.

37. *Nasal incisure at posterior end of mesorostral fossa*: (0) absent or small (does not extend posterior to supraorbital process); (1) deep and narrow (extends posterior to supraorbital process); (2) comparably deep but broad, with the anterior margin displaying a median convexity.

42. *Frontal roof*: (0) convex, more or less flat, or moderately concave between temporal crest (if latter present); (1) deeply concave or depressed overal (with or without a small median convexity) between temporal crests, but not sloping ventrad anteriorly; (2) deeply concave or depressed, and sloping steadily ventrad to anterior margin.

44. *Supraorbital process of frontal*: (0) not divided; (1) divided by one or more distinct, deep dorsoventral grooves indenting its lateral margin.

45. *Frontal roof*: (0) bear no knoblike bosses; (1) bears bilateral pair of knoblike bosses, more or less cylindrical in shape and directed anterad; or at least a distinct longitudinal ridge or swelling medial and parallel to, and distinct from, each temporal crest.

64. *Supraoccipital*: (0) wider in dorsal half than at ventral extremities of lateral borders; (1) wider ventrally than dorsally, or equally wide throughout.

66. *Exoccipitals*: (0) meet in a suture dorsal to foramen magnum; (1) do not meet in a suture.

72. *Hypoglossal foramen*: (0) surrounded by bone; (1) open (forming a notch), or absent.

77. *Processus retroversus of squamosal*: (0) absent: (1) present, moderately inflected; (2) present, not inflected; (3) present, strongly inflected.

85. *Ventral extremity of jugal*: (0) lies posterior to orbit; (1) lies approximately under posterior edge of orbit, but forward of jugal's postorbital process; (2) lies ventral to orbit.

88. *Preorbital process of jugal*: (0) relatively flat and thin (posteromedial-anterolateral breadth of portion lateral to maxillojugal suture > anteromedial-posterolateral thickness); (1) thick and robust (breadth ≤ thickness).

89. *Zygomatic process of jugal*: (0) as long as or longer than diameter or orbit; (1) shorter than diameter of orbit.

90. *Ventral rim of orbit*: (0) does not distinctively overhang lateral surface of jugal, where such a surface is present below the orbit; (1) does distinctly overhang.

97. *Posterior border of palatine*: (0) not incised, merely shallowly concave; (1) incised or deeply indented; (2) very deeply incised, to as far forward as level of M1.

101. *Alisphenoid canal*: (0) present; (1) absent.

102. *Pterygoid fossa*: (0) absent; (1) present, extending above level of roof of internal nares; (2) reduced, confined to below or above roof of internal nares.

103. *Foramen ovale*: (0) enclosed by bone; (1) opened to form a notch or incisure.

105. *Sphenopalatine region*: (0) long with pterygoid processes not enlarged; (1) short, with pterygoid process enlarged, thickened and downwardly projecting.

122. *Ventral border of horizontal mandibular ramus*: (0) straight or only slightly concave; (1) moderately concave, sharply downturned anteriorly; (2) moderately and evenly concave; (3) strongly concave.

123. *Accessory mental foramina*: (0) present, in addition to and usually posterior to the large principal foramen; (1) absent.

125. *Posterior border of mandible*: (0) descends ventrally or posteroventrally from condyle without marked interruption or abrupt change in direction; (1) bears a steplike process (processus angularis superior) below condyle; (2) has no distinct processus angularis superior but does have broadly convex outline beginning well below condyle.

128. *Horizontal ramus of mandible*: (0) slender (minimum dorsoventral height < 0.25 x length of mandible); (1) broad dorsoventrally (height ≥ 0.25 x length of mandible).

129. *Ventral border of horizontal ramus of mandible*: (0) tangent to angle; (1) not tangent to angle.

137. *First upper incisor*: (0) enamel crown distinct from root; (1) enamel extends entire length of tusk.

139. *First upper incisor*: (0) present; (1) vestigial or absent.

140. *Depth of I1 alveolus*: (0) much less than half of the premaxillary symphysis; (1) about half the length of the symphysis; (2) much greater than half the length of the symphysis.

141. *Cross section of I1 crown*: (0) suboval or subelliptical; (1) lens-shaped, with sharp anterior and posterior edges; (2) lozenge-shaped; (3) broad and extremely flattened mediolaterally.

142. *First upper incisor*: (0) with enamel on all sides, forming complete enamel crown; (1) with enamel mainly on medial side.

143. *Second and third upper incisors, first through third lower incisors*: (0) present, at least in part; (1) all absent.

144. *Canines*: (0) double-rooted; (1) single-rooted; (2) absent.

147. *Molar 3*: (0) with crown longer than or equal to that of M2; (1) smaller than M2; (2) with reduced circular crown.

157. *Permanent premolars*: (0) some double- or triple-rooted; (1) all single-rooted; (2) all absent.

**3. Matrix**

*Moeritherium* (Proboscidea)

0 0 1 0 0 0 1 ? 1 0 0 ? 0 0 0 0 0 ? ? 0 0 0 0 0 0 ? ? ? 0 0 1 0 0 ? ? 0 0 0 0 0 1 0 0

*Paleoparadoxia* (Desmostylia)

0 0 0 0 0 0 0 0 0 0 0 0 0 0 0 0 0 0 0 0 0 0 0 0 0 0 0 0 0 0 0 0 0 0 0 0 0 0 0 0 0 1 0

*Prorastomus sirenoides* (Prorastomidae)

1 0 0 1 1 1 1 0 ? 0 0 0 0 0 0 0 0 0 0 0 0 0 ? 0 0 0 1 0 0 0 0 0 0 0 ? 0 1 0 ? 0 0 0 0

*Ashokia antiqua* (Protosirenidae)

? 0 0 1 1 ? 1 0 0 0 0 0 0 0 0 0 0 0 0 0 0 0 0 0 ? 0 0 1 1 ? ? ? ? ? ? ? ? ? ? ? ? ? ?

*Miosiren kocki* (Trichechidae)

0 0 0 1 1 1 0 0 1 0 0 1 0 0 0 0 ? 0 ? 0 2 0 0 0 0 1 1 1 1 ? ? ? ? ? 1 0 2 0 0 1 2 2 1

*Eotheroides aegyptiacum*

? 0 0 1 1 1 0 0 0 0 ? 0 0 0 0 0 0 0 0 1 ? ? ? ? 0 1 1 1 ? 1 1 1 0 0 ? ? ? ? ? 0 1 0 1

*Halitherium taulannense*

2 0 0 1 1 1 ? 0 0 0 0 0 0 0 0 0 0 0 0 1 0 0 0 0 1 1 1 1 1 1 0 2 0 0 0 0 1 0 0 0 1 0 1

*Halitherium schinzii*

2 0 0 1 1 1 0 0 0 0 0 0 0 0 0 0 0 0 0 1 1 0 0 0 1 1 1 1 1 1 0 2 0 1 0 0 1 0 0 1 2 0 1

USNM 542417

2 0 0 1 1 1 0 0 0 0 0 0 0 0 0 0 0 0 0 1 1 0 0 0 1 1 1 1 1 3 1 2 1 1 ? 0 0 0 ? 1 2 0 2

*Metaxytherium krahuletzi*

2 0 0 1 1 1 1 0 0 0 0 1 0 0 0 0 1 0 0 1 1 0 0 0 1 1 1 1 1 3 1 2 1 1 0 0 0 0 0 1 2 0 2

*Metaxytherium medium*

2 0 0 1 1 1 1 0 0 0 0 1 0 0 0 0 1 1 0 1 2 0 0 0 1 1 1 1 1 3 1 2 1 1 0 0 0 0 0 1 2 0 2

*Metaxytherium serresii*

2 0 0 1 1 1 1 0 0 0 0 1 0 0 0 0 1 1 0 1 2 0 0 0 1 1 1 1 1 3 1 2 1 1 0 0 1 0 0 1 2 0 2

*Metaxytherium floridanum*

2 0 0 1 1 1 1 0 0 0 0 1 0 0 0 0 1 1 0 1 2 0 0 0 1 1 1 1 1 3 1 2 1 1 0 0 0 0 0 1 2 0 2

*Metaxytherium crataegense*

2 0 0 1 1 1 1 0 0 0 0 1 0 0 0 0 1 1 0 1 2 0 0 0 1 1 1 1 1 3 1 2 1 1 0 0 0 0 0 1 2 0 2

*Metaxytherium arctodites*

2 0 0 1 1 1 0 0 0 0 1 1 0 0 0 0 1 1 0 1 2 0 0 0 2 1 1 1 1 3 1 2 1 1 0 0 0 0 0 1 2 0 2

*Dusisiren jordani*

2 0 0 1 1 1 1 0 0 0 1 1 0 0 0 0 1 1 0 2 2 0 0 0 1 1 1 1 1 2 1 2 0 1 ? 1 0 ? ? 1 2 0 2

*Hydrodamalis cuestae*

2 0 0 1 1 1 1 1 0 0 2 1 0 0 0 0 0 1 0 2 2 0 0 0 1 1 1 1 1 2 1 2 0 1 ? 1 0 ? ? 1 2 ? 2

*Metaxytherium* sp. (UF 49051)

2 0 0 1 1 1 ? 0 0 0 0 1 0 0 0 0 1 1 0 1 1 0 0 0 1 1 1 1 1 3 ? 2 1 1 0 0 0 0 0 1 2 0 2

*Caribosiren turneri*

2 0 0 1 1 1 0 0 0 0 0 0 0 0 0 0 ? ? ? 1 ? 0 0 ? 1 1 1 1 1 ? ? ? ? ? ? 1 0 ? ? 1 2 0 2

*Crenatosiren olseni*

2 0 0 1 1 1 0 0 0 0 0 1 1 0 0 0 0 0 0 1 1 0 0 1 ? 1 1 1 1 3 1 2 1 1 0 0 1 0 0 1 2 0 2

*Nanosiren sanchezi*

2 0 1 1 ? 1 0 1 0 0 0 ? 1 1 ? 1 1 1 1 3 1 0 0 1 ? ? ? ? ? ? ? ? ? 1 0 0 0 0 0 1 2 0 2

*Nanosiren garciae*

2 ? ? 1 ? 1 0 1 0 0 0 ? 1 1 ? 1 1 1 1 3 1 0 0 1 ? 1 2 1 1 3 1 2 1 ? ? 0 0 ? ? 1 2 ? 2

*Dugong dugon*

2 0 0 1 1 1 0 1 1 0 0 1 2 2 1 1 1 1 0 3 2 1 0 1 1 1 1 1 1 3 1 2 1 1 1 0 2 0 1 1 2 0 2

*Bharatisiren indica*

2 1 0 1 1 1 0 0 0 0 0 3 1 0 1 0 0 0 ? 1 2 1 1 1 2 1 1 1 1 ? ? 2 ? ? ? 0 2 0 ? 1 2 0 2

*Bharatisiren kachchhensis*

2 1 0 1 1 2 0 0 0 1 0 0 1 2 1 0 1 0 ? ? 2 1 0 1 2 1 1 1 1 ? ? ? ? ? 1 0 2 0 ? 1 2 0 2

*Domningia sodhae*

2 2 0 1 1 1 0 0 0 0 0 3 1 2 0 0 0 0 ? ? 2 1 0 1 2 1 2 1 1 3 1 2 1 1 ? 0 2 1 ? 1 2 0 2

*Kutchisiren cylindrica*

2 1 0 1 1 1 0 0 0 0 0 1 1 2 0 0 1 0 0 1 2 1 ? 1 2 1 2 1 1 3 0 ? 1 ?1 0 2 2 1 1 2 0 2

*Dioplotherium manigaulti*

2 2 0 1 1 1 0 0 0 1 0 1 1 2 0 1 0 0 0 ? 2 1 1 1 2 1 2 1 1 ? ? ? ? ? 1 0 2 2 0 1 2 0 2

*Dioplotherium allisoni*

2 2 0 1 1 2 0 0 ? ? 0 3 1 1 1 1 0 0 0 1 2 1 1 1 1 1 2 1 1 3 0 2 1 1 1 0 2 2 1 1 2 0 2

*Dioplotherium* sp. (ECOCHM-2491)

2 2 0 1 1 2 0 0 0 0 0 3 1 1 1 0 0 1 0 1 2 1 1 1 ? 1 2 1 1 ? ? ? ? ? 1 0 2 2 1 1 2 0 2

*Xenosiren yucateca*

? 2 ? 1 ? ? ? 2 1 1 ? 3 1 2 0 1 ? ? ? 1 2 1 1 1 ? ? ? ? ? ? ? ? ? ? 1 0 ? 3 1 1 2 0 2

*Corystosiren varguezi*

2 ? 1 1 1 1 ? ? ? ? ? 3 2 2 0 1 ? 0 ? ? 2 1 0 0 ? ? ? ? 1 ? ? ? ? ? 1 0 2 3 1 1 2 0 2

*Rytiodus heali*

2 2 0 1 2 1 0 0 0 0 0 1 1 2 0 0 0 1 0 1 2 1 0 1 2 1 1 ? 1 3 1 ? 1 ? 1 0 2 3 1 1 2 0 2

*Rytiodus capgrandi*

? 1 1 1 1 ? ? 0 ? ? ? 1 1 2 1 0 ? 0 ? 1 3 ? 0 1 ? ? ? ? ? ? ? ? ? ? 1 0 2 3 1 1 2 0 2

**4. References**

1. Clementz MT, Sorbi S, Domning DP (2009) Evidence of Cenozoic environmental and ecological change from stable isotope analysis of sirenian remains from the Tethys-Mediterranean region. Geology 37: 307-310.

2. Domning DP, Sorbi S (2011) A new sirenian (Mammalia, Dugonginae) from the Miocene of Libya. J Vertebr Paleontol 31: 1338-1355.

3. Uhen MD, Coates AG, Jaramillo CA, Montes C, Pimiento C, Rincon A, Strong N, Velez-Juarbe J (2010) Marine mammals from the Miocene of Panama. J S Am Earth Sci 30: 167-175.

4. Toledo PM de, Domning DP (1991) Fossil Sirenia (Mammalia: Dugongidae) from the Pirabas Formation (Early Miocene), northern Brazil. Bol Museu Paraense Emílio Goeldi Sér Cienc da Terra 1: 119-146.

5. Domning DP (2001) Sirenians, seagrasses, and Cenozoic ecological change in the Caribbean. Palaeogeog Palaeoclim Palaeoecol 166: 27-50.

6. Domning DP (1978) Sirenian evolution in the North Pacific Ocean. University of California Publications in Geological Sciences 118: 1-176.

7. Aranda-Manteca FJ, Domning DP, Barnes LG (1994) A new Middle Miocene sirenian of the genus *Metaxytherium* from Baja California: relationships and paleobiogeographic implications. Proc San Diego Soc Nat Hist 29: 191-204.

8. Domning DP, Aguilera OA (2008) Fossil Sirenia of the West Atlantic and Caribbean region. VIII. *Nanosiren garciae*, gen. et sp. nov. and *Nanosiren sanchezi*, sp. nov. J Vertebr Paleontol 28: 479-500.

9. Sarko DK, Domning DP, Marino L, Reep RL (2010) Estimating body size of fossil sirenians. Mar Mammal Sci 26: 937-959.

10. Domning DP, Gingerich PD (1994) *Protosiren smithae*, new species (Mammalia, Sirenia), from the late Middle Eocene of Wadi Hitan, Egypt. Contrib Mus Paleontol, University of Michigan 29: 69-87.

11. Green EP, Short FT (2003) *World Atlas of Seagrasses*. Berkeley & California: University of California Press.

12. Domning DP (1994) A phylogenetic analysis of the Sirenia. Proc San Diego Soc Nat Hist 29: 177-189.

13. Bajpai S, Domning DP, Das DP, Vélez-Juarbe J., Mishra VP (2010) A new fossil sirenian (Mammalia, Dugonginae) from the Miocene of India. Neues Jahrb Geol P-A 258: 39-50.

14. Domning, DP, Gingerich PD, Simons EL, Ankel-Simons FA (1994) A new early Oligocene dugongid (Mammalia, Sirenia) from Fayum Province, Egypt. Contrib Mus Paleontol, University of Michigan 29: 69-87.
